# Physicochemical Aspects of the *Plasmodium chabaudi*-Infected Erythrocyte

**DOI:** 10.1155/2015/642729

**Published:** 2015-10-18

**Authors:** Eri H. Hayakawa, Seiki Kobayashi, Hiroyuki Matsuoka

**Affiliations:** ^1^Division of Medical Zoology, Jichi Medical University, Yakushiji 3311-1, Shimotsuke, Tochigi 329-0498, Japan; ^2^Department of Infectious Diseases, Keio University School of Medicine, 35 Shinanomachi, Shinjuku-ku, Tokyo 160-8582, Japan

## Abstract

Membrane electrochemical potential is a feature of the molecular profile of the cell membrane and the two-dimensional arrangement of its charge-bearing molecules. *Plasmodium* species, the causative agents of malaria, are intracellular parasites that remodel host erythrocytes by expressing their own proteins on erythrocyte membranes. Although various aspects of the modifications made to the host erythrocyte membrane have been extensively studied in some human *Plasmodium* species (such as *Plasmodium falciparum*), details of the structural and molecular biological modifications made to host erythrocytes by nonhuman *Plasmodium* parasites have not been studied. We employed zeta potential analysis of erythrocytes parasitized by *P. chabaudi*, a nonhuman *Plasmodium* parasite. From these measurements, we found that the surface potential shift was more negative for *P. chabaudi*-infected erythrocytes than for *P. falciparum*-infected erythrocytes. However, electron microscopic analysis of the surface of *P. chabaudi*-infected erythrocytes did not reveal any modifications as compared with nonparasitized erythrocytes. These results suggest that differences in the membrane modifications found herein represent unique attributes related to the pathogenesis profiles of the two different malaria parasite species in different host animals and that these features have been acquired through parasite adaptations acquired over long evolutionary time periods.

## 1. Introduction

Malaria, a serious infectious disease in humans, is caused by protozoan parasites of the genus* Plasmodium*. The parasite is widely distributed across tropical regions and affects large numbers of people living in Africa, South America, and Southeast Asia. Malaria infections are established by parasites released into the blood stream when parasite-infected female mosquitoes bite a vertebrate host; the consequent infection that follows occurs first in the liver followed by the erythrocytes. The clinical signs and symptoms of malaria infections in humans include chills, fever, body aches, headache, anemia, and spleen enlargement, while infections with* Plasmodium falciparum*, the most virulent, cerebral malaria-causing species [1], can be fatal. Infections with* Plasmodium* also induce cellular and molecular alterations to erythrocytes, such as cell adhesion [2–5], Band 3 clustering [6], erythrocyte-IgG association [7], increased hemichrome attachment to the host erythrocyte membrane [6], increased cell permeability [8], changes in erythrocyte rigidity [9–11], and, with certain parasite species, the appearance of knob-like structures on the cell surface [12–16]. These features of human malaria parasites are reported to vary in different hosts but are not known for all parasite species and hosts at the erythrocyte level.

*P. falciparum* is known to express a variety of proteins after it invades human erythrocytes. These proteins include the* P. falciparum* erythrocyte membrane protein-1 (PfEMP-1), knob-associated histidine-rich protein (KAHRP), and RIFIN, STEVOR, and SURFIN proteins amongst others, and these proteins are transported to the erythrocyte membrane. PfEMP-1, PfEMP-3, KAHRP are main components of the interconnect-protein complex known as “knobs” [17]; these proteins form a raised structure on the erythrocyte membrane surface and are considered to provide adhesion points for binding to endothelial cells [17–19]. Knobs on the erythrocyte membrane parasitized by* P. falciparum* have a characteristic morphology and uniform size (height: 18.2–25.3 nm [20]; diameter: ≈70 nm [21]). Also, some studies on monkey malaria infections have described parasite-infected erythrocytes with knob-like structures adhering to cells [14–16]. Furthermore, despite identification of furrows on the surface of the parasitized erythrocytes of* P. gallinaceum* (a chicken-infecting* Plasmodium* species), their function has not been extensively studied [22]. Expression of parasite-derived proteins on the erythrocyte membrane disturbs the balance of endogenous host proteins and their structural integrity [23–25]. Addition of new proteins induces protein-protein modifications and protein-lipid interactions at the erythrocyte membrane [25–28], as well as cytoskeleton remodeling, thereby resulting in changes in the net surface charge of the cell membrane.

Different species of malaria parasites infect humans, reptiles, birds, and rodents. One study has reported that chickens experience fevers, enlarged spleens, hemolysis, and other clinical signs when they become infected by avian malaria parasites [29]. In the case of rodent infections with malaria parasites of which there are three known species, one of the features of* P. berghei*-,* P. yoelii*-, and* P. chabaudi*-infected erythrocytes is their ability to sequestrate in various organs of the body [30–33]; however, the sequestration patterns differ from that of* P. falciparum*-infected erythrocytes [34, 35]. Despite some degree of overlap in the types of clinical and biological features of malaria infection in different host animals, there is insufficient information about the structural modifications made to host erythrocytes during malaria infections, particularly those caused by rodent malaria parasites. Nevertheless, differences do exist in the clinical features of infection with different species of* Plasmodium* in the same host and in different host animals. Hence, investigating the types of modification made to erythrocytes by different malaria parasite species in different host animals should further our understanding of malaria parasite biology. In this study, we investigated the physicochemical aspects of the erythrocyte membrane in terms of the structural modifications induced by infection with the rodent malaria parasite* P. chabaudi* and we describe here the host cell modifications that are characteristic of this parasite species.

## 2. Material and Methods

### 2.1. Erythrocyte Preparation and Malaria Parasite Infections

Animal infections were approved by “The Keio University Institutional Animal Care and Use Committee” and followed the “Institutional Guidelines on Animal Experimentation at Keio University.” Infection and erythrocyte collections were conducted as described in previous studies [36, 37]. Briefly, for the rodent malaria infections,* P. chabaudi* AS strain chloroquine sensitive was used. Four- to five-week-old female mice (BALB/c A Jc1, Nippon Bio-Supp. Center, Tokyo, Japan) were injected intravenously with 5 × 10^6^ parasitized erythrocytes. Mouse blood was collected when the parasitemia was ≈30–48% in ~1 mL of blood. The heparinized mouse blood was centrifuged at 300 ×g for 5 min to remove the buffy coat and washed twice with RPMI (Invitrogen Life Technologies, Grand Island, NY). Mature parasitized erythrocytes were separated using the MACS-LS Column system (Miltenyi Biotec K.K., Bergisch Gladbach, Germany) [38] for detergent-resistant membrane (DRM) fraction measurements. For the zeta potential measurements, whole mouse blood was used (mature* P. chabaudi*-infected erythrocytes were not separated from the uninfected erythrocytes because ~1 mL is the maximum volume that can be collected from a mouse heart after isoflurane (Mylan, Hertfordshire, UK) treatment and this amount is too small for measurement when only the parasitized erythrocytes were separated and used for the measurements).

Cultivation of* P. falciparum* was approved by the Bioethics Committee for Epidemiologic Research, Jichi Medical University (authorization number: 12–20), for clinical research with human blood samples. O^+^ human erythrocytes were purchased from the Japanese Red Cross Society (authorization number: 25J-0045).* P. falciparum* was cultured as described previously [39].* P. falciparum*-infected erythrocytes were separated by MACS Separators LS columns (Miltenyi Biotec K.K.) and then used for electron microscopy and zeta potential measurements or for obtaining the detergent-resistant membrane (DRM) fractions.

### 2.2. Transmission Electron Microscopy (TEM) and Scanning Electron Microscopy (SEM)

For TEM analysis, nonparasitized and parasitized erythrocytes were fixed for 60 min on wet ice in a mixture of 2% paraformaldehyde and 2% glutaraldehyde in HEPES buffer solution (pH 7.05) (Sigma-Aldrich Co. LLC, Saint Louis, MO) and then fixed for a further 60 min in 1% osmium oxide in the same buffer. The fixed specimens were dehydrated with stepwise concentrations of ethanol and then embedded in epoxy resin. Ultrathin sections were stained with uranyl acetate and lead citrate. Samples were examined with a JEM-1230 transmission electron microscope (JEOL, Tokyo, Japan). For the SEM analysis, erythrocytes were fixed for 2 hours on wet ice in 2% paraformaldehyde in 0.1 mol/L HEPES buffer (pH 7.4) The erythrocytes were then spread on a slide glass covered with MAS coating (Matsunami Glass Ind., Ltd., Kishiwada, Osaka, Japan). The samples were further fixed with 1% osmium oxide in phosphate-buffered saline (PBS, Invitrogen) for ~1 hour at room temperature and then dehydrated with ethanol. The samples were treated with isoamyl acetate (Wako, Osaka, Japan) and 100% ethanol (1 : 1 vol/vol) (Wako), dried using a critical point Dryer HCP-2 (Dryer HCP-2, Hitachi Koki Co., Ltd., Tokyo, Japan) for 5 min at 40°C, and then sputtered by an Ion Sputter (E-1030, Hitachi, Ltd., Tokyo, Japan) with Pt and Pd. Sample observations were performed using S-4300 scanning electron microscopy (Hitachi High-Technologies Corporation, Tokyo, Japan).

### 2.3. Measurement of Zeta Potentials

The surface potentials of erythrocyte membranes were measured by a zeta potential analyzer (Zeecom, Microtec, Co., Ltd., Funabashi, Japan) as described according to a previous study [40]. Measurements of parasitized mouse erythrocytes were conducted on whole mouse blood. Blood samples were washed twice with RPMI medium (Invitrogen) and then resuspended in a 10% volume in the same medium before the measurements were taken.

### 2.4. Collection of Detergent-Resistant Membrane (DRM) Fractions and Western Blotting

The effect of parasite infection on the lipid domain fraction of host erythrocytes was assessed by preparing lipid domain fractions according to an established method [27, 41]. Briefly, a 10 mL volume of packed erythrocytes was washed with RPMI (Invitrogen) twice; then a 200 *μ*L volume of the packed erythrocytes was thoroughly mixed with 800 *μ*L of ice cold 1% Triton X-100 (Sigma-Aldrich Co. LLC) in TBS buffer solution (Wako) and a proteinase inhibitor (Complete H; Roche Diagnostics, Mannheim, Germany) was included in each tube (total 1 mL). The samples were kept on ice for 20 min, after which they were each mixed with the same volume of 0.2 mol/L-Na_2_CO_3_ in 80% sucrose in TBS (final sucrose concentration 40% in a total volume of 2 mL). The 2 mL sample was transferred to a centrifugation tube (331372, Beckman Coulter, Inc., Brea, CA). Then, 6 mL of 30% sucrose in TBS was overlaid at 4°C, followed by an additional 3 mL of 10% sucrose in TBS as the upper layer. Ultracentrifugation was performed in a SW-41Ti rotor (Beckman Coulter) at 200,000 ×g for 18 h at 2°C and 300 *μ*L of each fraction was collected at 4°C and kept at −20°C.

To detect the DRM fraction and protein distribution, a NuPAGE 4–12% Bis-Tris Gel (Invitrogen) in MOPS SDS running buffer (Novex, Carlsbad, CA) was used for SDS-polyacrylamide gel electrophoresis. The separated proteins were transferred to a PVDF membrane using the iBlot Gel Transfer system (Invitrogen) and NuPAGE transfer buffer (Novex). Flotillin-1 was used as a reference raft marker and mouse anti-flotillin-1 was used as the monoclonal antibody against it (BD Biosciences, San Jose, CA). Next, the membrane was washed with 0.2% Tween 20 in PBS for 10 min (×3). The second antibody reaction was performed using peroxidase-conjugated AffiniPure Donkey Anti-Mouse IgG (H + L) (Jackson ImmunoResearch Laboratories, Inc., West Grove, PA). ECL Advance Western Blotting Detection Reagents (GE Healthcare Life Sciences, Pittsburgh, PA) were used as the developing reagents.

## 3. Results

### 3.1. Electron Microscopic Studies

**Figure 1:**
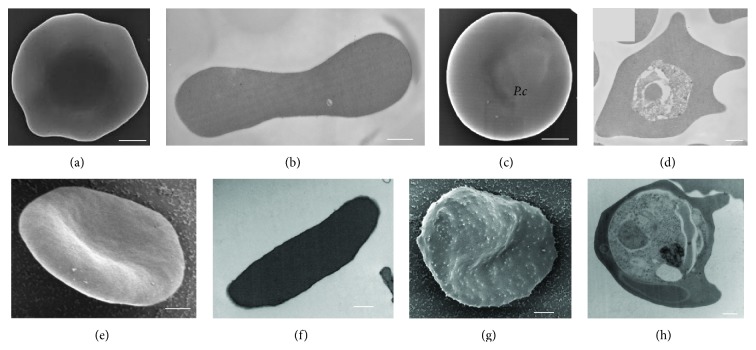
Nonparasitized mouse control erythrocyte (a, b). Electron micrographs of* P. chabaudi*-infected erythrocytes (c, d), showing the lack of any raised structures on the erythrocyte surface. In contrast, many knob-like structures on the surface of a* P. falciparum*-infected human erythrocyte are apparent (g). Electron micrograph illustrating the very smooth surface of a nonparasitized human erythrocyte (e). (a, c, e, g) SEM and (b, d, f, h) TEM. Bar = 1.0 *μ*m (a, c, e, g) and 500 nm (b, d, f, h).

Our EM images of nonparasitized mouse erythrocytes (Figures 1(a) and 1(b)) and* P. chabaudi*-infected mouse erythrocytes (Figures 1(c) and 1(d)) showed that the surface membrane was smooth and there were no structural modifications observed on the* P. chabaudi*-infected erythrocyte membrane surface (Figure 1(c)). Our results are in agreement with previous papers [42, 43]. A smooth surface in the* P. chabaudi*-infected erythrocytes was quite obvious in comparison with* P. falciparum*-infected erythrocytes, which form knob-like structures on the erythrocyte surface [44–47], unlike the very smooth surface topography of uninfected human erythrocytes observed in the scanning electron (Figure 1(e)) and transmission electron (Figure 1(f)) micrographs.

### 3.2. Zeta Potential Measurements and the Lipid Domain Fraction

**Figure 2:**
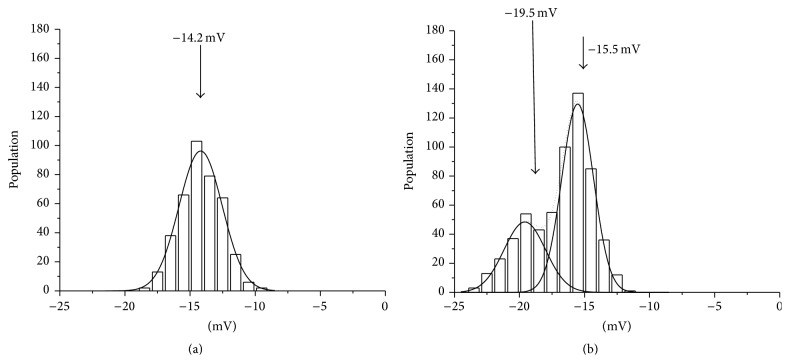
Z-potential data for nonparasitized (a) and* P. chabaudi*-parasitized (≈30–48% parasitemia) with nonparasitized (≈52–70%) (b) mouse erythrocytes. The membrane potential increased to a more negative value in the* P. chabaudi*-infected erythrocytes compared with the uninfected erythrocyte controls.

The zeta potential is an important index for evaluation of a material's surface in terms of its reflecting interfacial properties, dispersion, aggregation, and interaction with other materials. To investigate the properties of the erythrocyte membrane further, despite there being no obvious surface alterations to* P. chabaudi*-infected erythrocytes, we examined the zeta potential measurements for the surfaces of nonparasitized and* P. chabaudi*-infected erythrocytes. The electrochemical potential value obtained for* P. chabaudi*-infected erythrocytes shifted ≈25% to the negative side (voltage with an absolute value was induced) in comparison with the nonparasitized erythrocyte controls (Figures 2(a) and 2(b)). This tendency contrasts with that of the* P. falciparum*-infected erythrocyte membrane, where a shift of ≈26% to the positive side was observed (see Supplemental data 1 of the Supplementary Material available online at http://dx.doi.org/10.1155/2015/642729).

**Figure 3:**
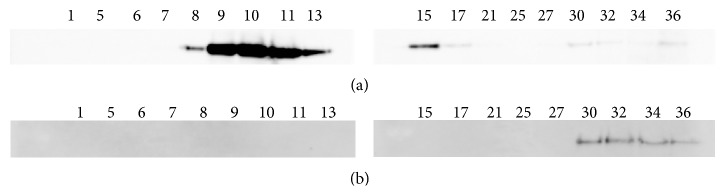
(a) and (b) represent a western blot comparison of the DRM fraction, which influences the raft domain, between nonparasitized and parasitized mouse erythrocytes, respectively. Flotillin-1 was used as a reference raft marker (MW = 48 kD).

To further characterize how parasite infection affected the properties of the erythrocytes, we examined the lipid domain fraction after sucrose gradient density centrifugation separation. This fraction influences the raft domain in the nonparasitized and parasitized erythrocyte membrane. Anti-flotillin-1 was used as a marker for raft/DRM. With the nonparasitized mouse erythrocytes, the lipid domain was present in the upper-to-middle and lower fractions, and most fractions existed in the upper fraction (Figure 3(a)). In contrast, the lipid domain was present only in the bottom layer in the parasitized erythrocytes (Figure 3(b)) and the lipid domain was not present in the upper layers. These results show that* P. chabaudi* altered the distribution of the lipid domain and the interaction between lipids and proteins in the erythrocyte membrane, even though the surface structure of the erythrocyte was not altered by the presence of the parasite.

## 4. Discussion

### 4.1. The Effect of Malaria Parasite Infections on the Topography, Surface Charge, and Lipid Domain of Host Erythrocytes

Generally, lipids are asymmetrically distributed between the inner and outer bilayer membranes of the erythrocyte with phosphatidylserine and phosphatidylethanolamine existing in the inner membrane and phosphatidylcholine in the outer membrane. Various studies have shown that not only malaria parasites modify the erythrocyte membrane surface structure, but also the internal structure and properties of the infected erythrocytes are modulated after invasion. In particular, infection with* P. falciparum* results in significant alteration of the erythrocyte surface topography, with alteration of the inner structure of the parasitized erythrocyte [39], alteration to protein transportation [46, 48], modifications to the erythrocyte cytoskeleton [47], and changes in the lipid distribution [25] and the inner microstructure of the erythrocyte membrane [25, 49]. In the present study, the Z-potential values were altered in* P. chabaudi*-infected erythrocytes (Figures 2(a) and 2(b)), even though* P. chabaudi* does not modulate the erythrocyte membrane topography (Figure 1(c)). The surface potential charge of the infected erythrocytes showed a “net” charge for the erythrocyte surface. Thus, our data also suggest that some properties of the parasitized erythrocyte membrane should be altered by infection with* P. chabaudi*. Evidence for this possibility is that the DRM fraction pattern changed between the nonparasitized and parasitized erythrocytes (Figures 3(a) and 3(b)). The direction of shift in the potential charge for* P. chabaudi*-parasitized mouse erythrocytes was opposite to that of the shift observed for* P. falciparum*-parasitized human erythrocytes (Supplemental data 1). We found no direct evidence of an alteration in erythrocyte topology or a change in the erythrocyte surface under the influence of* P. chabaudi* infections. However, our data suggest that parasite-encoded proteins, such as knob-like structures on erythrocytes, possibly play a role in altering the surface potential charge on the erythrocyte by altering the distribution of cytoskeleton proteins and/or lipid-protein interactions.

Zeta potential is an electrochemical aspect of a particle's surface, and information about a particle's dispersibility, aggregability, and adhesion ability can be obtained from this measurement. If zeta potential takes a cross value around zero, the repulsive force between particles becomes weak and the particles will eventually aggregate. This physicochemical aspect corresponds to the phenomenon of* P. falciparum* erythrocyte adhesion (Supplemental data 1). Our data showed a shift away from a zero point for* P. chabaudi*-infected erythrocytes; however, the absolute shift values for* P. chabaudi*- and* P. falciparum*-infected erythrocytes were in a similar range and the shift range did not depart far from the zero point. Parasitized erythrocytes exhibit similar phenomena to aging erythrocytes compared with normal erythrocytes, such as increased levels of hemichrome, higher levels of oxidization, alterations in Band 3 aggregation and the cytoskeleton, and changes in protein expression related to immune function, adhesion, and permeability of the erythrocyte [6, 50].

*P. chabaudi*- and* P. falciparum*-infected erythrocytes are reported to have properties that promote different patterns of sequestration [33, 42, 51, 52] in their respective host species. Indeed,* P. chabaudi*-infected erythrocytes are reported to sequester in the lungs and liver of mice but do not adhere to the mouse brain, which is the cause of cerebral malaria in* P. falciparum* infections in humans. These differences in the behavior of* P. chabaudi*-infected mouse erythrocytes and* P. falciparum*-infected human erythrocytes are consistent with our zeta potential data. One possible explanation for the different pattern of sequestration observed between* P. falciparum*- and* P. chabaudi*-infected erythrocytes is that the* var* multigene family of* P. falciparum*, which is a component of knobs and is necessary for sequestration of* P. falciparum*-infected erythrocytes, does not exist in* P. chabaudi* [53].

Zeta potential provides information on the “total surface charge” of an erythrocyte, and this parameter is modulated by complexes formed between different proteins, the lipid bilayer, and the erythrocyte cytoskeleton. We do not know which proteins or protein complexes reduce the absolute value of the surface charge of* P. falciparum*-infected erythrocytes. However, knob-associated proteins are one of the candidates for the difference observed in the zeta potential data between* P. falciparum*- and* P. chabaudi*-infected erythrocytes. Taken together, our data are consistent with previous reports and physiological evaluation of parasite-infected erythrocytes.

### 4.2. Evolution of Malaria Parasites and Their Animal Hosts

Humans have been exposed to* P. falciparum* malaria infections for over 4,000 years [54], but different species of malaria parasites have been parasitizing other mammals such as rodents as well as avian and reptilian hosts for much greater evolutionary time periods [55–57]. Malaria parasites of humans survive in their hosts by invading nonnucleated erythrocytes. However, birds have nucleated erythrocytes making it unclear why this type of erythrocytes is preferred by some* Plasmodium* spp. Furthermore, the role of the furrow-like structure observed in* P. gallinaceum*-infected erythrocytes from chicken [22] is also not clear. Here, we have shown that* P. chabaudi* rodent malaria parasites do not modulate the surface of the parasitized erythrocyte (Figure 1, Table 1).* P. chabaudi*-infected erythrocytes have been reported to undergo sequestration in the lungs and liver but do not sequestrate in the brain, spleen, or other tissues, unlike* P. falciparum*-infected erythrocytes [35]. One possible explanation for this is that* P. chabaudi* does not possess the* var* multigene family (as discussed in the previous paragraph) that encodes* P. falciparum* PfEMP-1; thus,* P. chabaudi*-infected erythrocytes probably do not express PfEMP-1, neither do they express KAHRP, which together form the components of knobs. Hence, rodent parasite-infected erythrocytes do not become trapped in the capillary blood vessels of the brain and rodents infected with this parasites species are not subject to the lethal effects of cerebral malaria. This feature of infection with* P. chabaudi* parasites allows them to survive for longer time periods in their hosts and was possibly selected for over the course of parasite evolution.

Coevolution between some malaria parasites and their hosts has occurred over very long evolutionary time periods, during which time these parasites have developed strategies that enable them to survive for long time periods in their respective host animals. The differences in the* P. chabaudi*- and* P. falciparum*-infected erythrocytes that we observed in this study may be one of the ways in which these parasites have adapted to their respective host animals (rodents and humans). Parasites eventually evolve to not kill their hosts but coexist with them.

## Supplementary Material

Supplemental data 1 Z-potential of (a) Non-parasitized human erythrocytes and (b) P. falciparum-infected erythrocytes. The absolute value of the membrane potential is reduced in the parasitized erythrocytes.

## Figures and Tables

**Table 1:** Comparison of *P. falciparum*- and *P. chabaudi*-infected host erythrocytes.

	Human/*P. falciparum *	Mouse/*P. chabaudi *
Morphological change	+	−
Zeta potential (numerical value)	Reduced	Induced
Parasitemia	+	++
Anemia	+	++
