# Supplementary figures and images for: Physicochemical Aspects of the Plasmodium chabaudi-Infected Erythrocyte
Source: Biomed Res Int. 2015 Oct 18;2015:642729. doi: 10.1155/2015/642729 (PMC4628737; doi:10.1155/2015/642729)

## Supplemental data 1

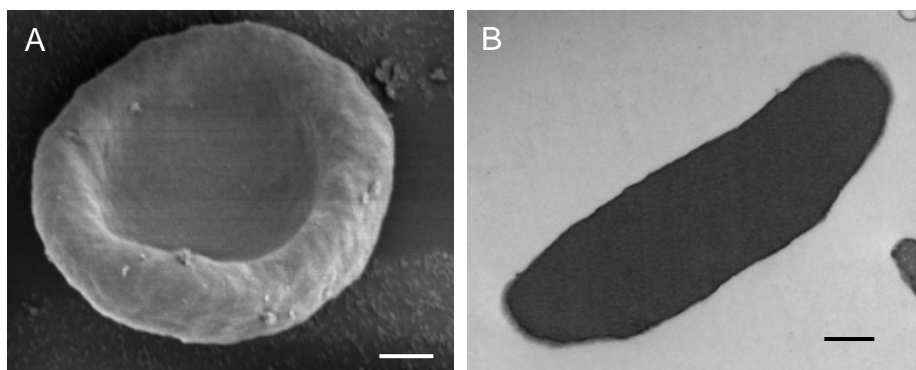

## Supplemental data 2

(a) Human erythrocytes

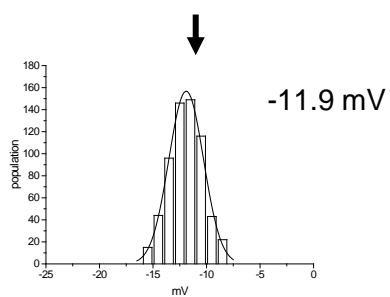

(b) with *P. falciparum*

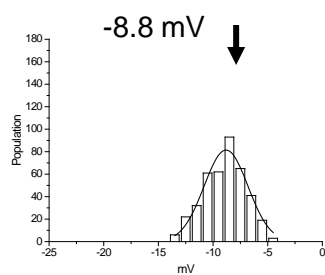

Supplement: Supplementary file 1 — Supplemental data 1 Z-potential of (a) Non-parasitized human erythrocytes and (b) P. falciparum-infected erythrocytes. The absolute value of the membrane potential is reduced in the parasitized erythrocytes. [file 642729.f1.pdf]
